# Advancing prostate cancer detection: a comparative analysis of PCLDA-SVM and PCLDA-KNN classifiers for enhanced diagnostic accuracy

**DOI:** 10.1038/s41598-023-40906-y

**Published:** 2023-08-23

**Authors:** Priya Dubey, Surendra Kumar

**Affiliations:** Electrical and Electronics Engineering, Birla Institute of Technology, Ranchi, Jharkhand 835215 India

**Keywords:** Computational biology and bioinformatics, Engineering

## Abstract

This investigation aimed to assess the effectiveness of different classification models in diagnosing prostate cancer using a screening dataset obtained from the National Cancer Institute’s Cancer Data Access System. The dataset was first reduced using the PCLDA method, which combines Principal Component Analysis and Linear Discriminant Analysis. Two classifiers, Support Vector Machine (SVM) and k-Nearest Neighbour (KNN), were then applied to compare their performance. The results showed that the PCLDA-SVM model achieved an impressive accuracy rate of 97.99%, with a precision of 0.92, sensitivity of 92.83%, specificity of 97.65%, and F1 score of 0.93. Additionally, it demonstrated a low error rate of 0.016 and a Matthews Correlation Coefficient (MCC) and Kappa coefficient of 0.946. On the other hand, the PCLDA-KNN model also performed well, achieving an accuracy of 97.8%, precision of 0.93, sensitivity of 93.39%, specificity of 97.86%, an F1 score of 0.92, a high MCC and Kappa coefficient of 0.98, and an error rate of 0.006. In conclusion, the PCLDA-SVM method exhibited improved efficacy in diagnosing prostate cancer compared to the PCLDA-KNN model. Both models, however, showed promising results, suggesting the potential of these classifiers in prostate cancer diagnosis.

## Introduction

Globally, prostate cancer ranks among the primary causes of cancer-related mortality in men. It is a critical public health issue requiring accurate diagnostic procedures for prompt detection and treatment. However, current methods for diagnosing prostate cancer, such as the Prostate-Specific Antigen (PSA) screening test and the Digital Rectal Exam (DRE), have limitations that necessitate further investigation.

However, current diagnostic techniques such as the Prostate-Specific Antigen (PSA) screening test and Digital Rectal Exam (DRE) have limitations, leading to false positives and over-diagnosis. The dramatic difference in survival rates between benign and aggressive prostate tumors underscores the need for reliable diagnostic tools to enhance patient outcomes^1–3^.

Given the alarming statistics pertaining to prostate cancer, it is evident that accurate diagnostic methods are essential for effective treatment and enhanced patient outcomes. Prostate cancer is the second most common cause of death in men over the age of 65. It is anticipated that there will be around 0.25 million new instances of prostate cancer in the United States alone, with a 13% mortality rate within the next year^4^. Globally, it is expected that approximately 27% of the estimated 1.4 million cases of prostate cancer would result in mortality^5^.

It is critical to emphasize the dramatic difference in survival rates between benign and aggressive prostate tumors. While benign cases have a five-year survival rate of 100%, malignant patients have a far lower survival rate of only 31%^6,7^. This highlights the important need for reliable diagnostic tools capable of distinguishing benign from malignant instances, allowing for earlier targeted therapies to enhance patient outcomes.

To address this need, researchers have access to diverse datasets from various academic sources and scientific databases such as PubMed and Google Scholar. Some of these datasets are well organized and structured but most of them are unstructured and needs data pre-processing and data standardization^8^. The real-world dataset that contains information on screening results may be used to acquire the prostate results and examine the variance in gland size and how they may aid in disease diagnosis. A variety of these datasets are prone to different issues, including the following:

- Some of the datasets have traits that are particularly significant in cancer identification, however, the majority of the benchmark datasets do not. These properties are absent from the majority of the datasets. Take, for instance, the size-sag and size-trans variables in the PLCO dataset^9^. Most of the datasets that are available today don’t pay much attention to these attributes.
- Since the pre-processed standard datasets are often of a hypothetical character, the results of any research that is carried out on them are not applicable in the actual world.
- Only a handful of the datasets that may be accessed contain absolutely no missing data or only a very minute portion of it. Real-world medical data that is currently available does not support this assumption. Because of this, the strategies that were used to fix this problem are quite different across the different datasets.

In light of the challenges mentioned earlier, the primary objective of this research is to address the limitations in prostate cancer screening datasets by employing various data pre-processing methods. The goal is to create a standardized dataset that includes essential characteristics relevant to prostate cancer diagnosis. Moreover, the study aims to investigate the interdependency among these features, understanding how they collectively contribute to accurate classification.

In prostate cancer research, one well-known dataset is the Prostate, Lung, Colorectal, and Ovarian (PLCO) Cancer Screening Trial^10^. However, initial screening datasets often suffer from the “curse of dimensionality,” containing an extensive number of characteristics that can adversely affect model performance. To overcome this challenge, dimension reduction techniques, such as PCLDA, are applied to reduce the number of features while preserving their discriminative power.

AI-based solutions have been at the forefront of addressing complex challenges in various domains, including healthcare. In retinal disorders, cancer detection, finger vein recognition, and other fields, AI has revolutionized the way we approach diagnosis and decision-making^11–18^. These AI-based approaches have shown great promise in improving diagnostic accuracy and efficiency, allowing for automated detection and classification of diseases with high precision.

Drawing from the advancements in AI and combining them with dimension reduction techniques like PCLDA, this research aims to develop a robust and accurate classification model for prostate cancer. The PCLDA-based model, along with SVM and KNN classifiers, classifies prostate results into three classes: negative (Class 1), abnormal-suspicious (Class 2), and abnormal-non-suspicious (Class 3). By integrating AI into prostate cancer diagnosis, the study seeks to achieve earlier detection of aggressive cases and provide personalized treatment recommendations for better patient outcomes. Below is a summary of the significant contributions of this research:

- In this research work, a novel PCLDA-based classification model is developed to extract the most significant features from the acquired dataset.
- A standardized dataset is provided that may be used by researchers in the further data processing.
- Both the PCLDA-SVM and PCLDA-KNN classification model are based on one-vs-one classification thus resulting in multiple classes that are: negative, suspicious and abnormal non-suspicious
- The evaluation of each model’s performance on 57,698 participants suggests that the suggested strategy has excellent potential for generalization.

The following is the article’s structure: In “Introduction” section of this paper , a concise overview is provided about the PCa biomarkers and the data processing procedures. “Related works” section gives a brief review on the related works. All of the characteristics of the datasets and the methodology used are explained in “Methodology” section. The discussion and analysis of the results can be found in “Result and discussion” section, and the conclusion can be found in “Conclusion” section.

## Related works

In the realm of medical data analysis, numerous approaches have been explored and refined to ensure accurate and reliable results. The preprocessing of medical data plays a pivotal role in optimizing the performance of machine learning and deep learning algorithms, particularly in the context of diagnosis and classification tasks. In recent years, there has been a remarkable surge in the adoption of advanced techniques in this domain.

One notable study conducted by Bilal et al. showcased the effectiveness of various classification techniques in addressing specific medical challenges. For instance, they employed Binary Tree, Support Vector Machine (SVM), and k-Nearest Neighbors (KNN) algorithms to classify and detect Diabetic Retinopathy with an impressive accuracy of 98.06%^11,12^. Moreover, they leveraged the power of Convolutional Neural Networks (CNN) for lung nodule detection, demonstrating the potential of deep learning in this critical area of medical imaging analysis^15,17^.

Jenny and Preetha^19^ used a method that combines Principal Component Analysis (PCA) and Linear Discriminant Analysis (LDA) to get rid of noise and get rid of features that didn’t belong in the breast cancer dataset. Alshareef et. al. used a logarithmic transformation to turn an asymmetrical prostate cancer dataset into a symmetrical one so that appropriate results could be obtained through statistical testing. They used a technique called scaled variance to normalize the dataset, replacingany values in the dataset with their average value. They used a filter called the flat pattern filter, which eliminates genes to make the dataset that is used for studying biologically meaningful phenomena easier to work with^20^.

Boluwaji et.al. developed an SVM based early detection model for prostate cancer with an accuracy of 90% and sensitivity of 94%. They performed on Kaggle datasets and used the PCA technique for feature reduction and then compared the SVM-PCa result with that of Logistic Regression^21^.

Adiwijaya et al.^22^ performed PCA feature reduction on the DNA microarray data along with SVM and backpropagation classifiers resulting in an accuracy of 94.98% and 96.07% accuracies, respectively.

Some researchers applied KNN with eight features and Decision tree (DT) Classifiers on histopathology images of prostate cancer and showed the KNN method had better accuracy of 84.44% with 100% sensitivity and specificity^23^.

Some of the research works focuses on detecting diabetic retinopathy using U-Net, transfer learning, weighted filters, and grey wolf optimization. These AI-based technologies have the potential to improve the accuracy and efficiency of identifying diabetic retinopathy from fundus images, allowing for earlier intervention and vision loss prevention^13,14^.

Another study uses neuro-optimization to optimize numerical models for HIV infection therapy, which could lead to better drug discovery and personalized treatment regimens^16,18^. These papers highlight the expanding importance of AI in biomedical research, offering promising tools to improve medical diagnosis and patient outcomes.

## Methodology

We acquired data from the Cancer Data Access System (CDAS) project of the National Cancer Institute (NCI) with PLCO id 934^24^. Every methodology employed in this study was completely adhered to the applicable standards and regulations. The National Cancer Institute (NCI) carefully reviewed the project proposal and approved it, ensuring that it met established research criteria. To increase data quality and usability for analysis, the unstructured dataset was preprocessed. First, inefficiencies and inconsistencies in the dataset were identified. Then, to standardize all variables, we employed data normalization. To minimize dimensionality, the researchers employed PCLDA, which combines PCA and LDA. To maximize dataset variance, PCA transforms variables into uncorrelated principal components. This reduces dimensionality while retaining the majority of the dataset’s information. LDA maximizes the separation of dataset classes. By projecting data into a lower-dimensional space, LDA improves categorization. The PCLDA approach decreases the dimensionality of a dataset while retaining discriminating information. To minimize data dimensionality and improve discriminating, PCLDA combines PCA and LDA.

### Data description

**Figure 1:**
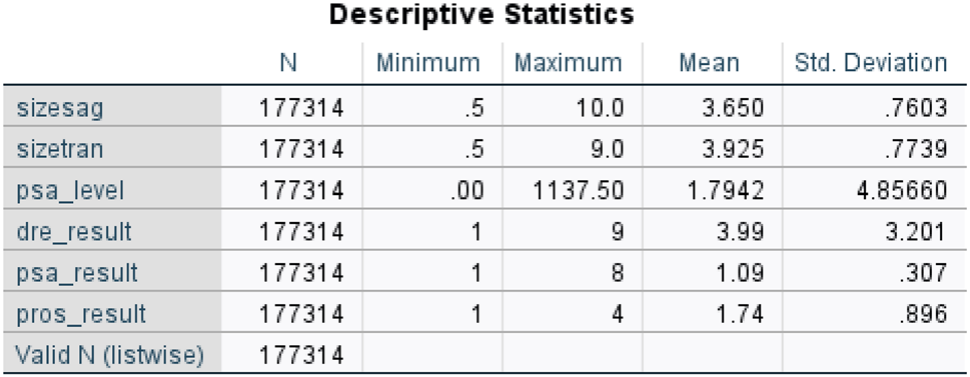
Statistical description of prostate cancer dataset.

**Figure 2:**
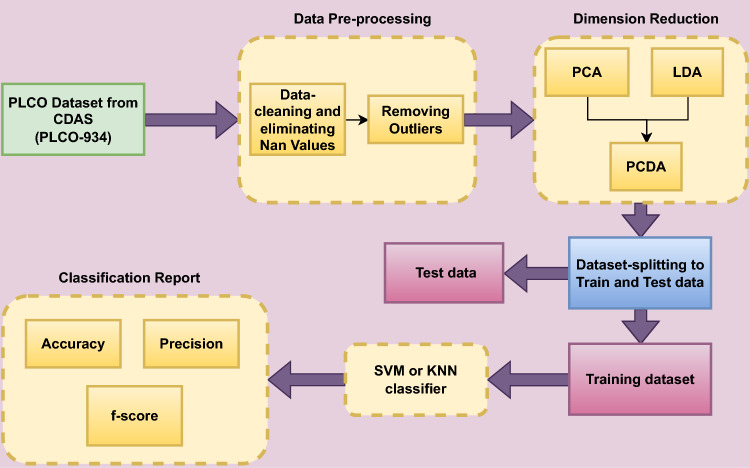
Flow diagram for proposed methodology.

The PLCO dataset consisted of 177,314 entries and 80 columns. These selected features included PSA and DRE cancer screening results, blood draw results, QA DRE results, explanations for insufficient tests, and any further abnormalities that were not malignant. Table 1 shows the data attributes for screening datasets and their descriptions. Figure 1 gives the statistical information about important features of data (Fig. 2).

**Table 1:** Data attributes and their description.

Attribute name	Values	Description	Text
dre pvis1	1–4	Days between randomization and DRE screening	$1 \rightarrow$ The first visit; $2 \rightarrow$ Second visit and so on
dre ref	1–4	Referral status for dre	$1 \rightarrow$ Significant Abnormality, $2 \rightarrow$ Moderate Abnormality, $3 \rightarrow$ Slight Variation from Normal, $4 \rightarrow$Normal
find enlrg	1	Additional enlargement information	$1 \rightarrow$ Yes
prospalp	0–1	Prostate palpability	$0\rightarrow$ No, $1 \rightarrow$ Yes
sizesag	0.5–8	Sagittal gland size	Numeric
sizetran	0.5–9	Transverse gland size	Numeric
psa level	0–1137.5	PSA level recorded for screening	Numeric
dre result	1–9	DRE screening result	$1\rightarrow$Negative(NG), $2\rightarrow$ Abnormal, suspicious (AS), $3 \rightarrow$ Abnormal, non-suspicious (ANS), $4\rightarrow$Inadequate screen (IN), $8 \rightarrow$ Not done, expected, $9 \rightarrow$ Not done, not expected”
psa result	1–8	PSA screening result	$1 \rightarrow$NG, $2 \rightarrow$ AS, $4\rightarrow$ IN, $8 \rightarrow$ Not done
pros result	1–4	Combined prostate screening result	$1 \rightarrow$ NG, $2 \rightarrow$ AS, $3\rightarrow$ ANS, $4 \rightarrow$ IN

### Data pre-processing

The Screening dataset undergoes an initial step of data pre-processing to ensure its quality and integrity. Null values, also known as missing values, can hinder accurate analysis and interpretation of the data. Therefore, appropriate measures are taken to address these null values based on the specific feature being considered. These null values are handled by either removing them entirely from the dataset (if null values > 70%) or replacing them with a suitable value (i.e. mean or max value) that reflects the overall characteristics of the feature.

By employing these data pre-processing techniques, the Screening dataset becomes more suitable for subsequent analysis, enabling reliable insights to be derived from the refined data.

The data pre-processing involves cleaning the data, dealing with the NaN values and removing outliers in the following ways:

- *data cleaning* This stage involves the management of NaN values in datasets. This may be accomplished in various ways, including removing these numbers or replacing them with the most frequent values, the mean value, or the standard deviation. The minimum values of their respective attributes are substituted for NaN values in PLCO datasets.
- *Removing Outliers* Each characteristic is box plotted to identify outliers. The removal of these points further cleans the data. The dataset is then normalised using the min–max approach.

**Figure 3:**
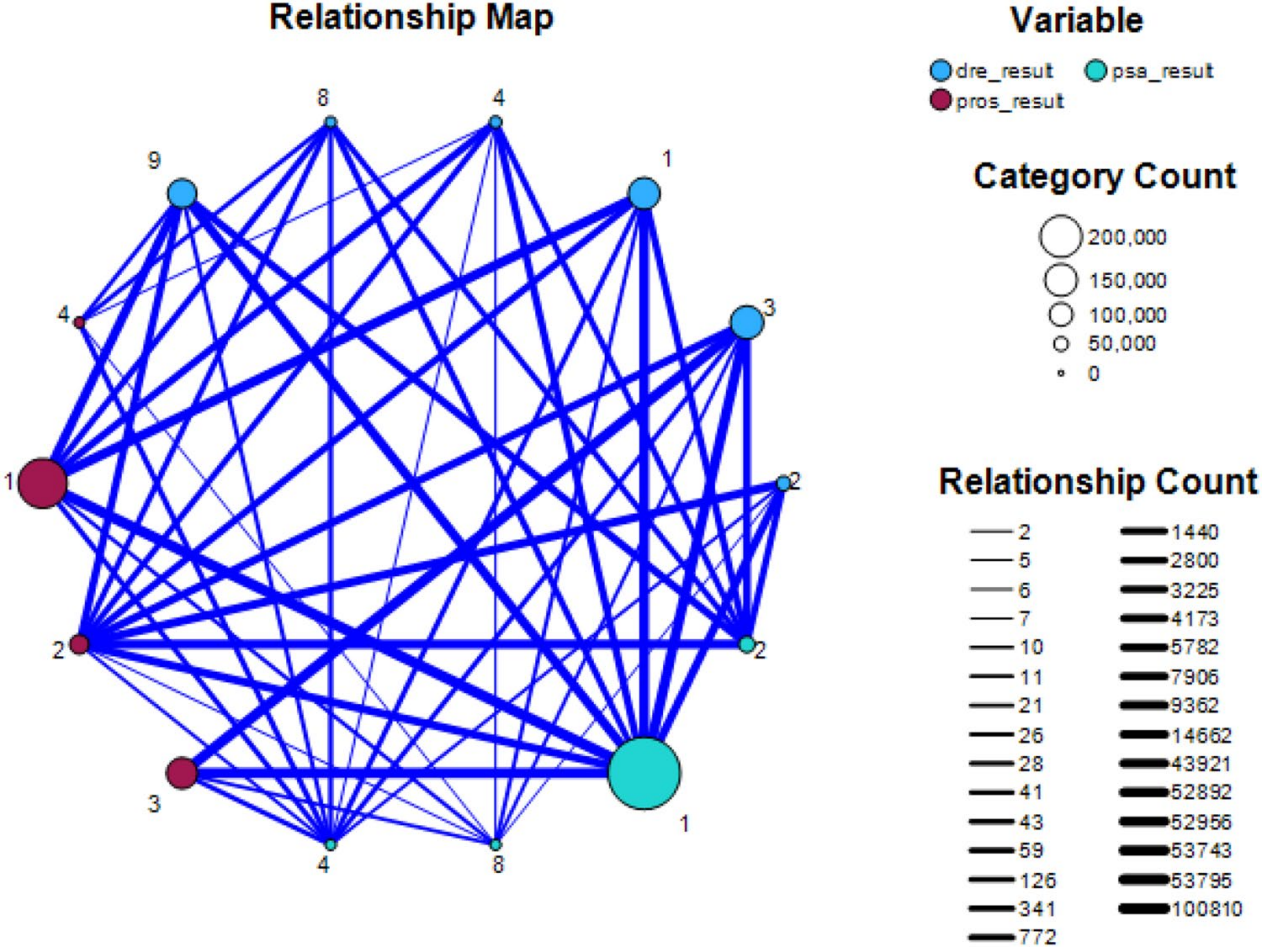
Relationship map between the variables psa result, dre result and pros result.

### Statistical tests

**Figure 4:**
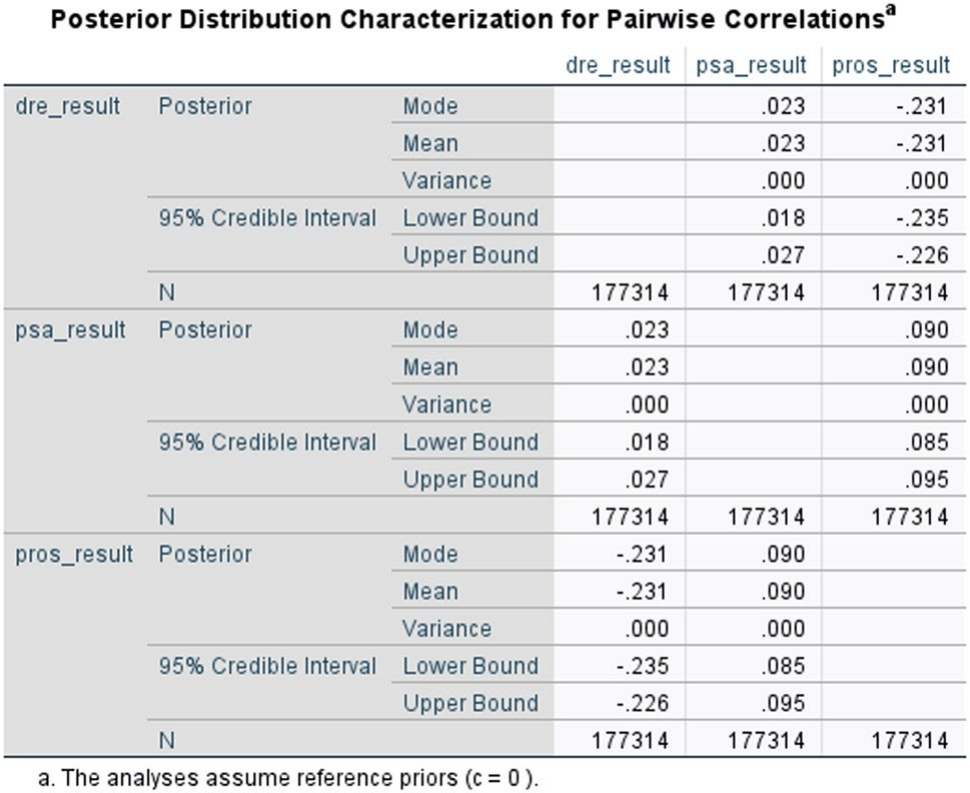
Bayesian correlation between psa result, dre results and pros results.

Understanding the relationships between variables is crucial in extracting meaningful insights from datasets. This is done by performing some standard statistical test on the data. First of all, a relationship map that visually represents the correlations between the variables (Fig. 3). This map serves as a graphical tool to identify patterns and potential connections among the ’psa result’, ’dre result’, and ’prostate result’. Next, Bayesian correlation is computed to understand the strength and direction of the relationship between these variables (Fig. 4).

**Figure 5:**
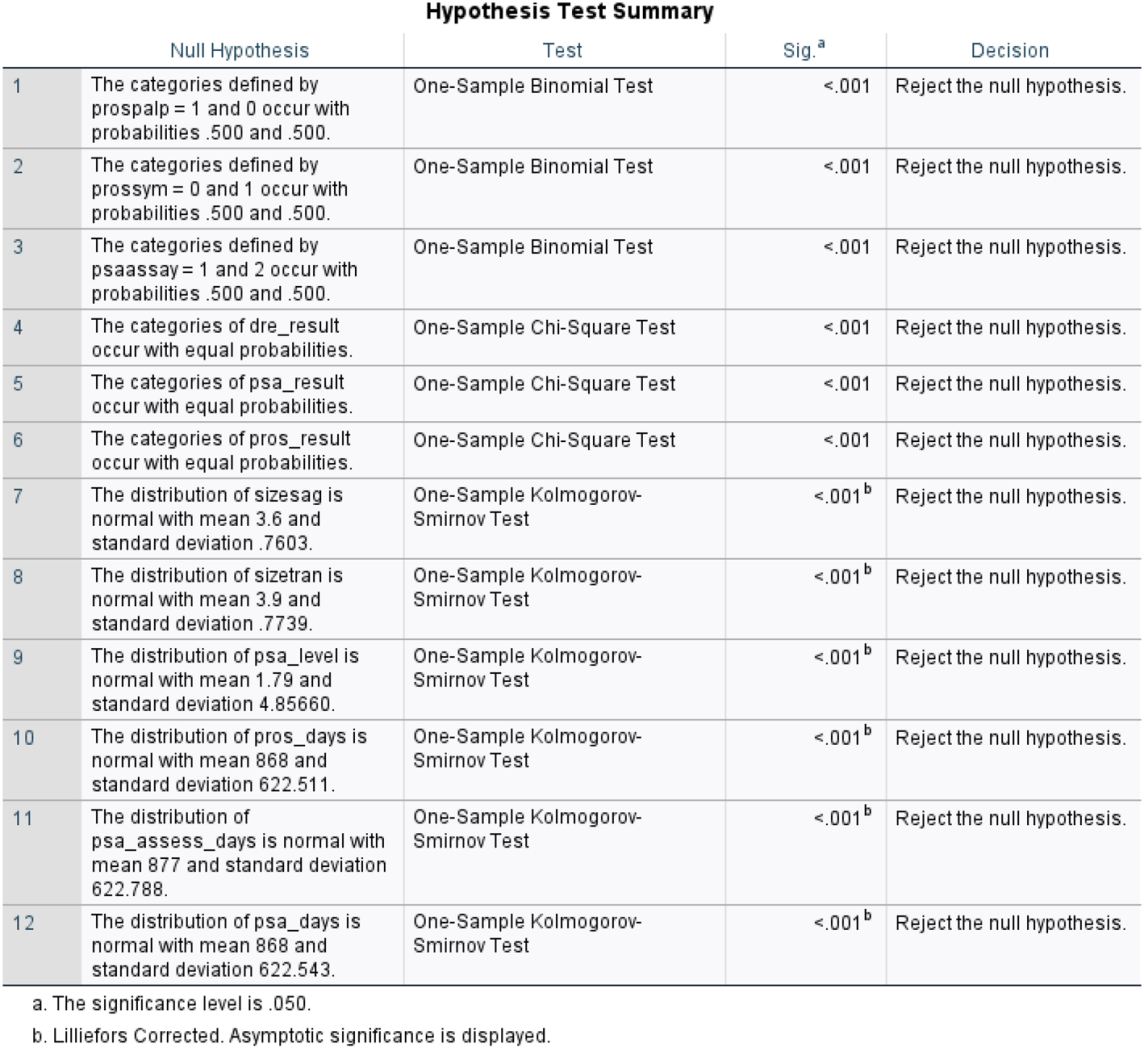
Non-parametric test results for prostate cancer dataset.

**Figure 6:**
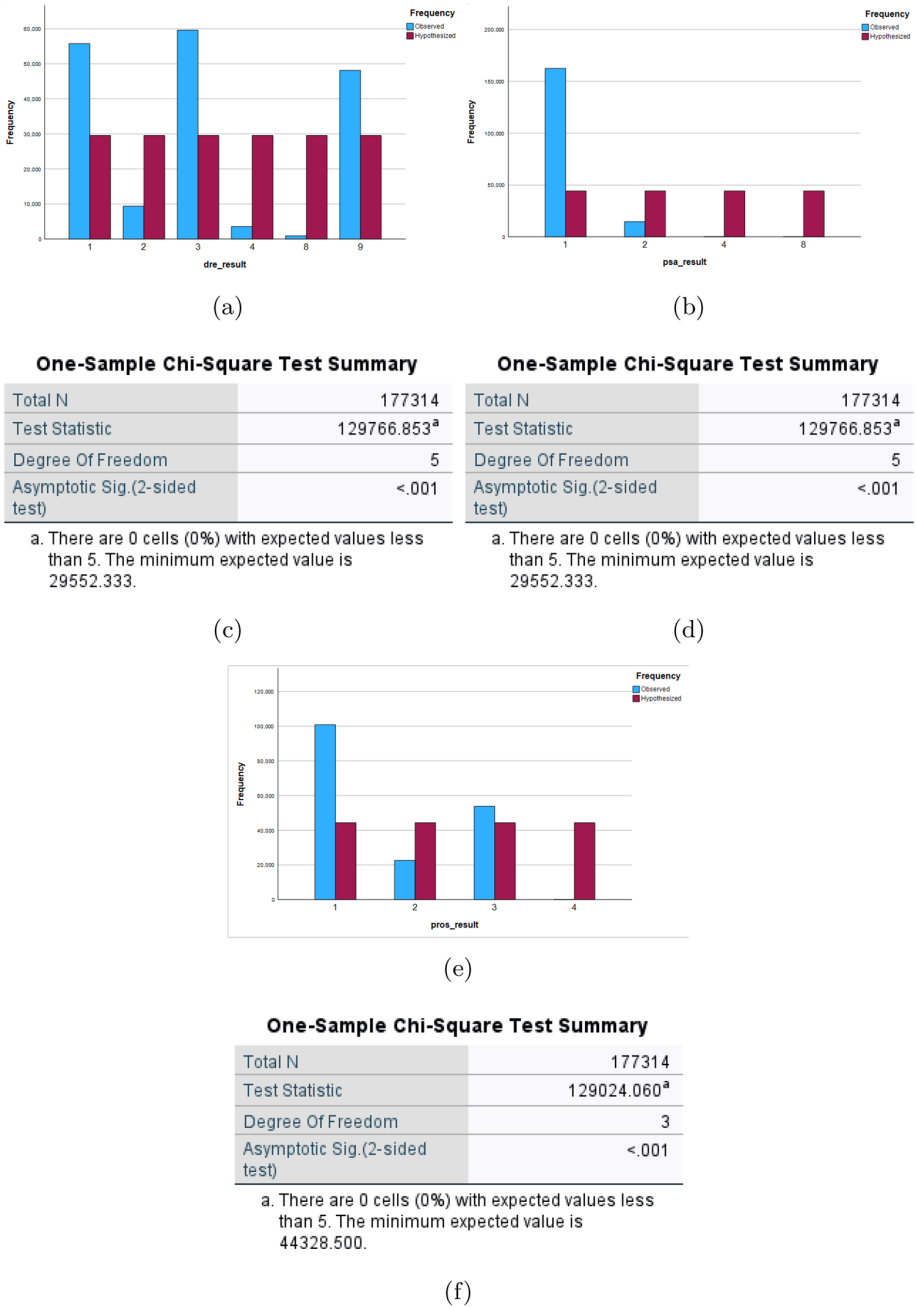
Plot using Chi-square tests: (**a**–**c**) shows the frequency plots and (**d**–**f**) are the tabular results for dre results, psa results and pros results respectively.

**Figure 7:**
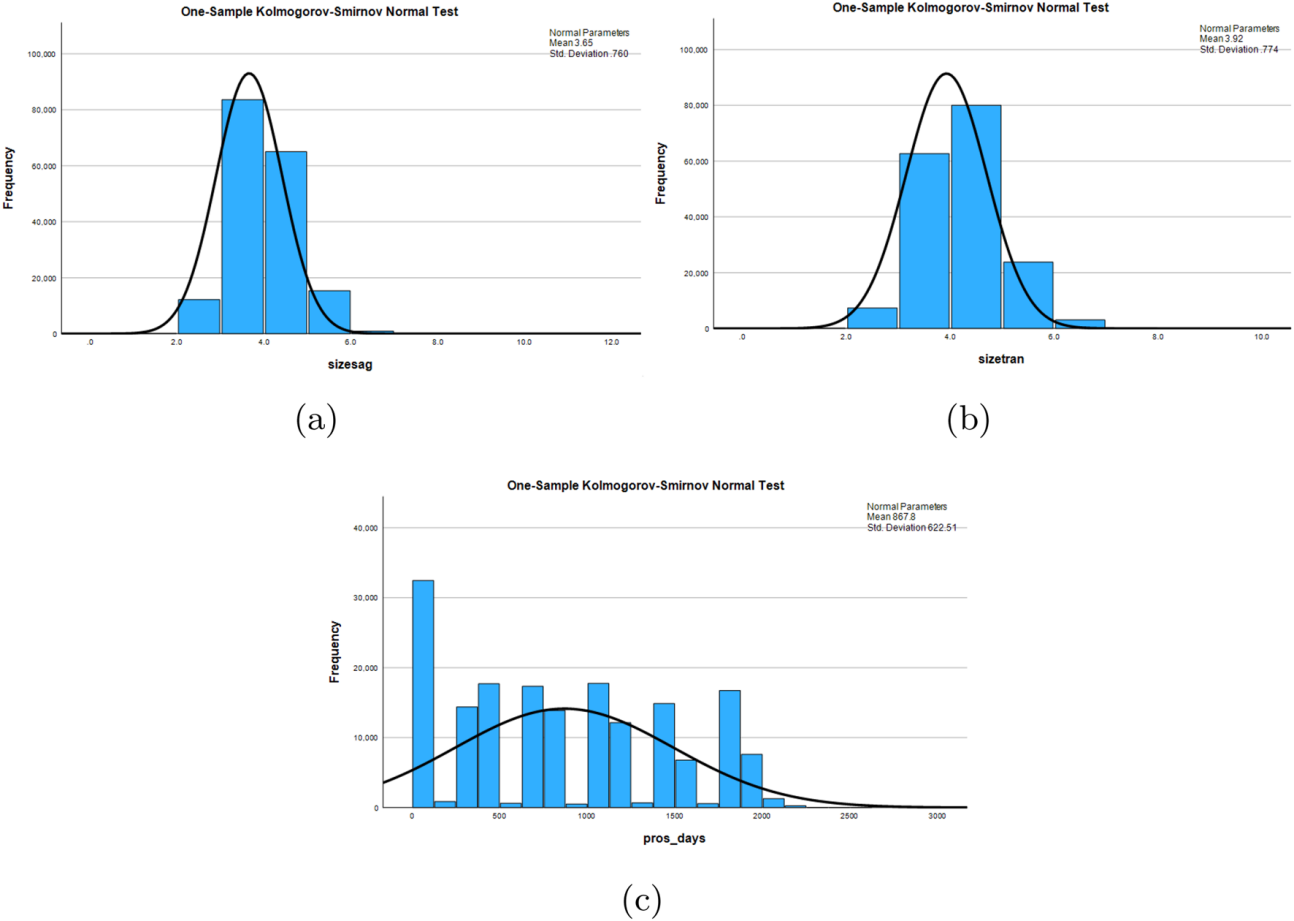
Plots using one-sample Kolmogorov–Smirnov test.

Different non-parametric tests are performed on the resulting dataset and are summarized in Fig. 5. One-sample Chi-square test results and one-sample Kolmogorov–Smirnov test results are shown in Figs. 6 and 7 respectively. The one-sample chi-square test determines if the observed and predicted frequencies in a categorical data sample differ significantly. The chi-square test compares observed frequencies to anticipated frequencies to evaluate if there is evidence to reject the null hypothesis and conclude that the categorical variable’s distribution differs significantly. A non-parametric statistical test called the Kolmogorov–Smirnov test evaluates whether a sample follows a given probability distribution or if two samples are derived from the same distribution.

Another non-parametric test, called Friedman test is applied to determine if there is a significant inter dependency between the psa result, dre result, and prostate result variables. The null and alternate hypothesis are defined as:

${\textbf {H}}_{\textbf {O}}$: There is no significant relationship or dependence between the psa result, dre result, and prostate result variables.

${\textbf {H}}_{\textbf {A}}$: There is a significant relationship or dependence between the psa result, dre result, and prostate result variables. The *p* value obtained was 0.0 with test statistics of 167424.0381697389. The test statistic indicates the overall level of difference among the variables, while the *p* value assesses the statistical significance of these differences. The obtained *p* value of 0.0 suggests strong evidence to reject the null hypothesis of no difference among the variables.

### Features extraction and dimension reduction

After the dataset has been pre-processed, the important features are extracted from it. These characteristics include the PSA level, the findings of the DRE, the prostate result, as well as the sagittal size and transverse size of the prostate glands. After that, the Standard scaler method from the NumPy library is used to adjust the values of these features.

To handle the initial dataset’s large dimensionality, which consisted of 80 columns, various dimension reduction techniques are employed. Specifically, three approaches, namely Principal Component Analysis (PCA), Linear Discriminant Analysis (LDA), and PCLA (a fusion between PCA and LDA), are utilized. These techniques aid in obtaining a more comprehensive understanding of the extensive dataset. PCA identifies the principal components that capture the maximum variance in the data, allowing for a lower-dimensional representation. LDA, on the other hand, focuses on finding a projection that maximizes class separability, thus facilitating better discrimination between different classes. PCLA combines the strengths of both PCA and LDA, leveraging their complementary aspects to achieve improved dimension reduction and enhanced class separation^25,26^. The resulting datasets is then separated into test data and training data. A classifier is then given the training and testing data to categorize the prostate findings (i.e. pros results). There are three classes: class 1 for prostate test findings that are negative, class 2 for suspicious results, and class 3 for abnormalities that are not suspicious. Figure 2 illustrate the phases of the suggested technique.

#### Principal component analysis (PCA)

Principal component analysis is a feature reduction methodology that recognizes correlations and patterns in a dataset so that it may be translated into a much lower-dimensional dataset without losing crucial information^27,28^. Figure 9 represents the steps involved in PCA technique.

The first stage is the normalisation of the data, followed by the evaluation of the covariance matrices. Covariance matrices, given by **C**, illustrate the connection between variables in a dataset.

$$\begin{aligned} C=\left[ \begin{array}{cccccccccc} 1.00 &{} 0.019 &{} -0.016 &{} -0.011 &{} 0.002 &{} 0.032 &{} 0.014 &{} 0.005 &{} 0.1 &{} 0.096\\ 0.019 &{} 1.00 &{}0.09&{} 0.072 &{} 0.063 &{} -0.048 &{} -0.102 &{} 0.120&{} -0.123 &{} -0.123 \\ -0.016 &{} 0.09 &{} 1.00 &{} 0.741 &{} 0.08 &{} 0.002 &{} 0.148 &{} 0.18 &{} 0.104 &{} 0.103 \\ -0.011 &{} 0.072 &{} 0.741 &{} 1.00 &{} 0.087 &{} -0.002 &{} 0.143 &{} 0.198 &{} 0.100 &{} 0.099 \\ 0.002 &{} 0.064 &{} 0.08 &{} 0.088 &{} 1.00 &{} -0.013 &{} -0.002 &{} 0.301 &{} -0.022 &{}-0.022 \\ 0.032 &{} -0.048 &{} 0.002 &{} -0.002 &{} -0.013 &{} 1.00 &{} 0.33 &{} -0.035 &{} 0.321 &{} 0.321 \\ 0.014 &{} -0.102 &{} 0.148 &{} 0.143 &{} -0.002 &{} 0.329 &{} 1.00 &{} -0.019 &{} 0.840 &{} 0.840 \\ 0.005 &{} 0.120 &{} 0.18 &{} 0.198 &{} 0.301 &{} -0.035 &{} -0.019 &{} 1.00 &{} -0.051 &{} -0.047 \\ 0.096 &{} -0.123 &{} 0.104 &{} 0.100 &{} -0.022 &{} 0.321 &{} 0.839 &{} -0.051&{} 1.00 &{} 0.999 \\ 0.096 &{} -0.123 &{} 0.103 &{} 0.099 &{} -0.022 &{} 0.321 &{} 0.839 &{} -0.04&{} 0.999 &{} 1.00\\ \end{array}\right] \end{aligned}$$

It is essential to identify variables with a high degree of dependency since they include misleading and redundant information that reduces the model’s overall performance. The eigenvectors and their respective eigenvalues are then assessed and placed in decreasing order. The eigenvector with the greatest eigenvalues is the most significant and is the first Principal Component. The eigenvalues are given by $e_i$s:

$$\begin{aligned} e_i=\left[ \begin{array}{cccccccccc} 3.97 &{} 1.89 &{} 1.174 &{} 1.006 &{} 0.926 &{} 0.850 &{} 0.682 &{} 0.225 &{} 0.258 &{} 0.00065\\ \end{array}\right] \end{aligned}$$

**Figure 8:**
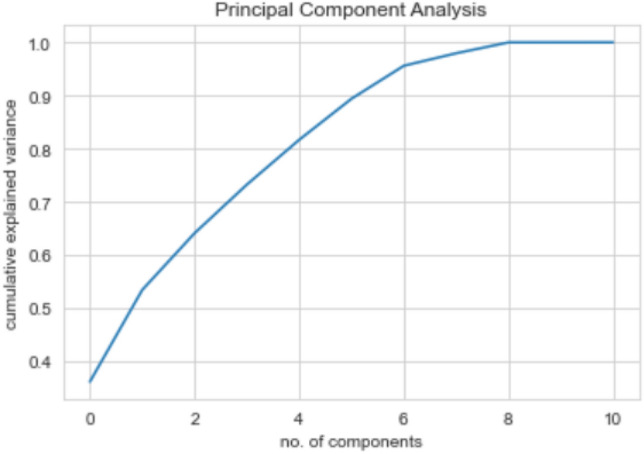
Cumulative Variance Ratio of Principal Components: The increasing ratio indicates the amount of information captured by the principal components.

The cumulative variance is visualized by plotting it against the Principal Components (PCs) (see Fig. 8), illustrating that the first PC exhibits the highest variance, followed by the second PC, and so on. This plot emphasizes the progressive decrease in variance as we move towards higher-order PCs, highlighting the significance of the initial PCs in capturing the maximum amount of variance in the dataset. The algorithm 1 summarises these stages.

**Figure 9:**
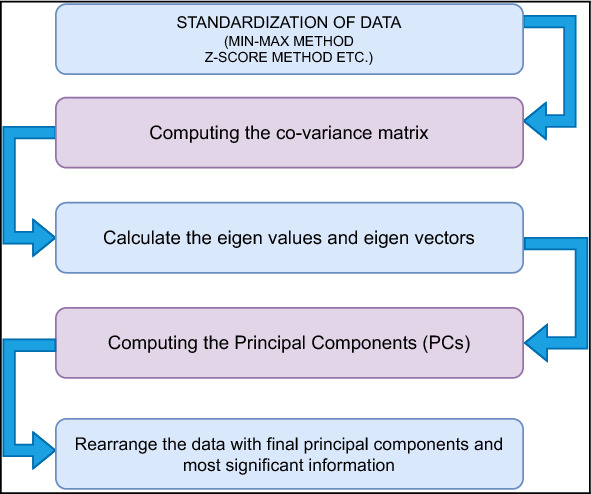
PCA process.

**Figure 10:**
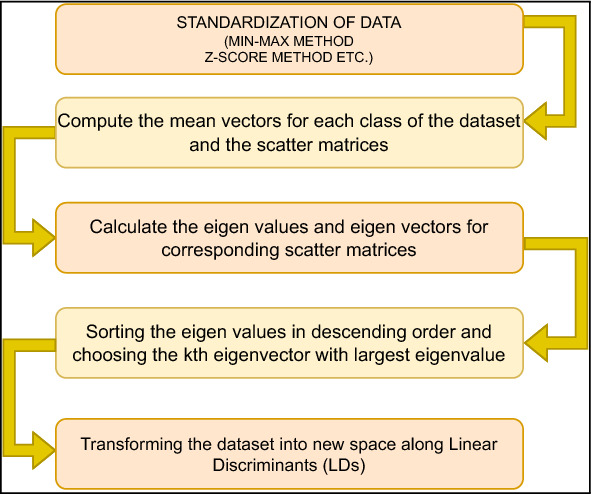
LDA process.

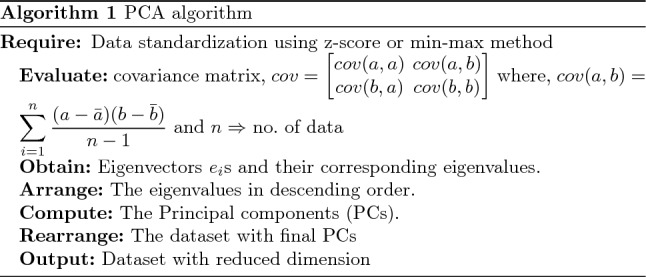



The PCA technique successfully reduced the dimensionality of the data to 10 dimensions. In order to further enhance the class separability, the next step involves applying the Linear Discriminant Analysis (LDA) technique, as illustrated in “ Linear discriminant analysis (LDA)” section. LDA aims to maximize the separation between different classes by finding a linear projection that maximizes the between-class variance and minimizes the within-class variance. This step will help uncover discriminative features that contribute significantly to the classification task, leading to improved class separability and potentially enhanced performance in categorizing the prostate findings (Fig. 9).

#### Linear discriminant analysis (LDA)

The objective is to project a dataset onto a lower-dimensional space with adequate class separability to prevent overfitting. The general strategy for LDA is quite comparable to that of a Principal Component Analysis; however, instead of looking for the component axes that maximize the variance of our data, we are interested in finding the axes that maximize the separation between multiple classes. Figure 10 and algorithm 2 summarizes the step involved in LDA. Here, $S_i$ is the scatter-matrix for *i*th class, $S_W$ is the for within class and $S_B$ for between-class.
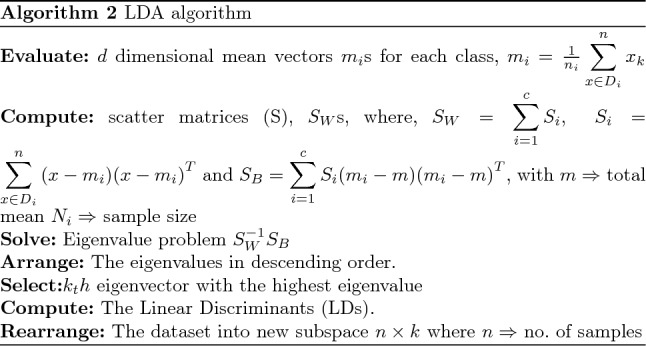


The Linear Discriminants, represented by the vector, *LDs*:

$$\begin{aligned} \left[ \begin{matrix}-0.5 &{} 2.5 &{} 2.7 &{} -0.3 &{} -1.09 &{} -0.26 &{} -3.1 &{} -0.2 &{} -0.28 &{} -15.6\\ \end{matrix}\right] \end{aligned}$$

The absolute values of the coefficients in the vector of linear discriminant might be taken into account to identify the most significant linear discriminant. The feature or feature combination that contributes most to class separation is indicated by the linear discriminant with the largest absolute value.By projecting the data onto this discriminant, the class separability is improved, enabling more effective classification of the prostate findings.

#### Splitting dataset: training and testing data

**Figure 11:**
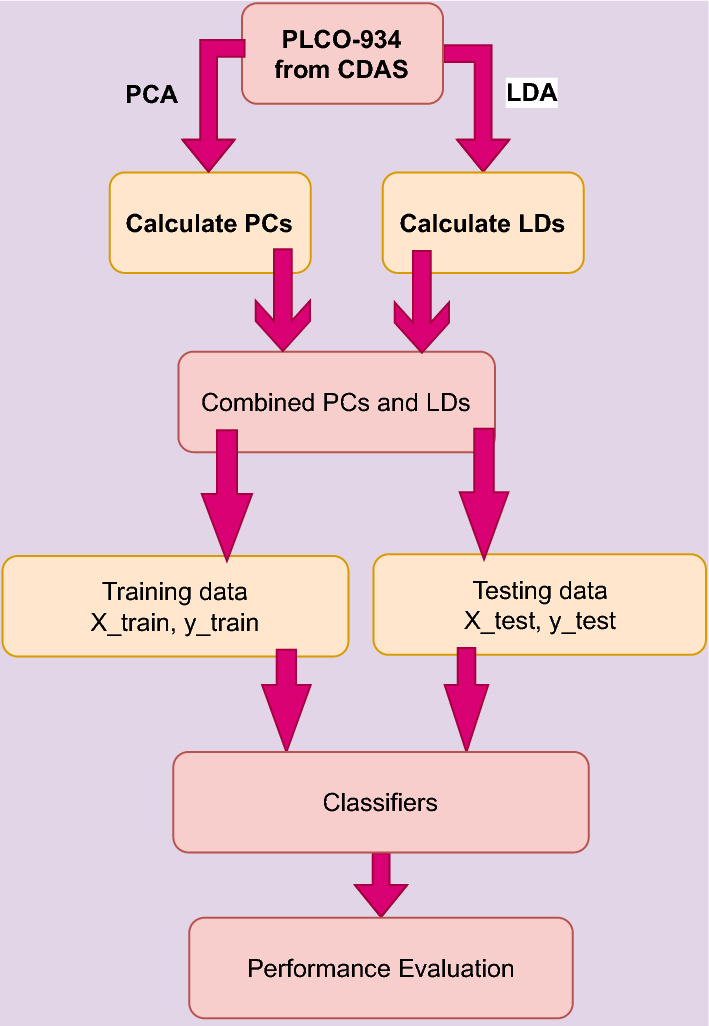
Data splitting into training and testing data.

After obtaining the PCs and LDs, they are joined for the PCLDA method. The datasets are divided into training and testing data: the training dataset, which accounts for 80% of the data, and the testing dataset, which accounts for the remaining 20%. When PCs and LDs are joined, a new feature space is created, which is then fed into the training and testing stages. Figure 11 shows the data splitting process.

#### Classification model based on SVM and KNN

Our module for classification consists of two categorization processes: the training and testing phases. During the training phase, the model was trained by applying an input feature set extracted using PCA and LDA transformation models to the model’s built-in SVM^29^and KNN classifiers with k $=$ 5.

*SVM*

SVM is often used as a supervised machine learning technique for multivariate classification for separating the two data groups. The classification of data into different classifications requires a hyperplane. This can be mathematically expressed as:

1 $$\begin{aligned} W^Tx-b=0 \end{aligned}$$

where *W* represents weight vectors, namely $W = \{w_1, w_2, w_3, \ldots ,w_n\}$; *n* represents the number of features; and $\frac{b}{||{w}||}$ denotes the offset to hyperplane. Tow hyper planes $H_1$ and $H_2$ are selected such that they satisfy:

2 $${} H_1: W^Tx-b=1; \text {everything above this line belongs to one class}$$

3 $${} H_2: W^Tx-b=-1; \text {everything below this line belongs to other class}$$

For *i*th point, using Eqs. 2 and 3, The following inequalities hold:

4 $${} W^Tx_i-b\ge 1; \text {if} \quad y_i=1$$

5 $${} W^Tx_i-b\le -1; \text {if} \quad y_i=-1$$

We get the optimization problem using Eqs. 4 and 5 :

6 $$\begin{aligned} y_{i}(w^Tx_{i}-b)\ge 1 \forall i \in {1, \ldots n} \end{aligned}$$

Due to its exceptional performance in handling high-dimensional data and its ability to mitigate overfitting, the Support Vector Machine (SVM) algorithm has proven to be an invaluable tool in prostate cancer screening. SVM demonstrates its prowess by effectively handling datasets with numerous features, which is particularly relevant in cancer screening scenarios that involve considering multiple biomarkers. Moreover, SVM’s capability to handle imbalanced datasets is particularly advantageous when dealing with cancer screening data characterized by unequal class distributions.

To optimize the SVM classifier, we embarked on an extensive hyperparameter tuning process. Initially, we employed the default hyperparameters provided by the SVM algorithm. Subsequently, we employed the GridSearchCV method, a powerful technique for hyperparameter optimization, to further enhance the SVM model’s performance. The optimal hyperparameters identified through GridSearchCV were ’C’: 100, ’gamma’: 1, resulting in a best score of 0.9834. These hyperparameters were specifically chosen to enhance the SVM model’s performance on our prostate cancer screening dataset.

*KNN*

KNN is one of the non-parametric machine learning algorithms. This classifier stores training instance data since constructing a Generalised Internal model is challenging. At each breakpoint, categorization values are calculated using the clear majority. For each data class, the query point given indicates the highest value inside k-NN. The classifier calculates the euclidean distance $d_i$s between training data points $x_i$s. These distances are then sorted in ascending order. The first positive *k* and their corresponding points are found. The data point *x* belongs to the *i*th class if the following equation holds true:

7 $$\begin{aligned} k_i > k_j \forall i \ne j \quad \text {then} \quad x \in i_{th} \text {class} \end{aligned}$$

**Figure 12:**
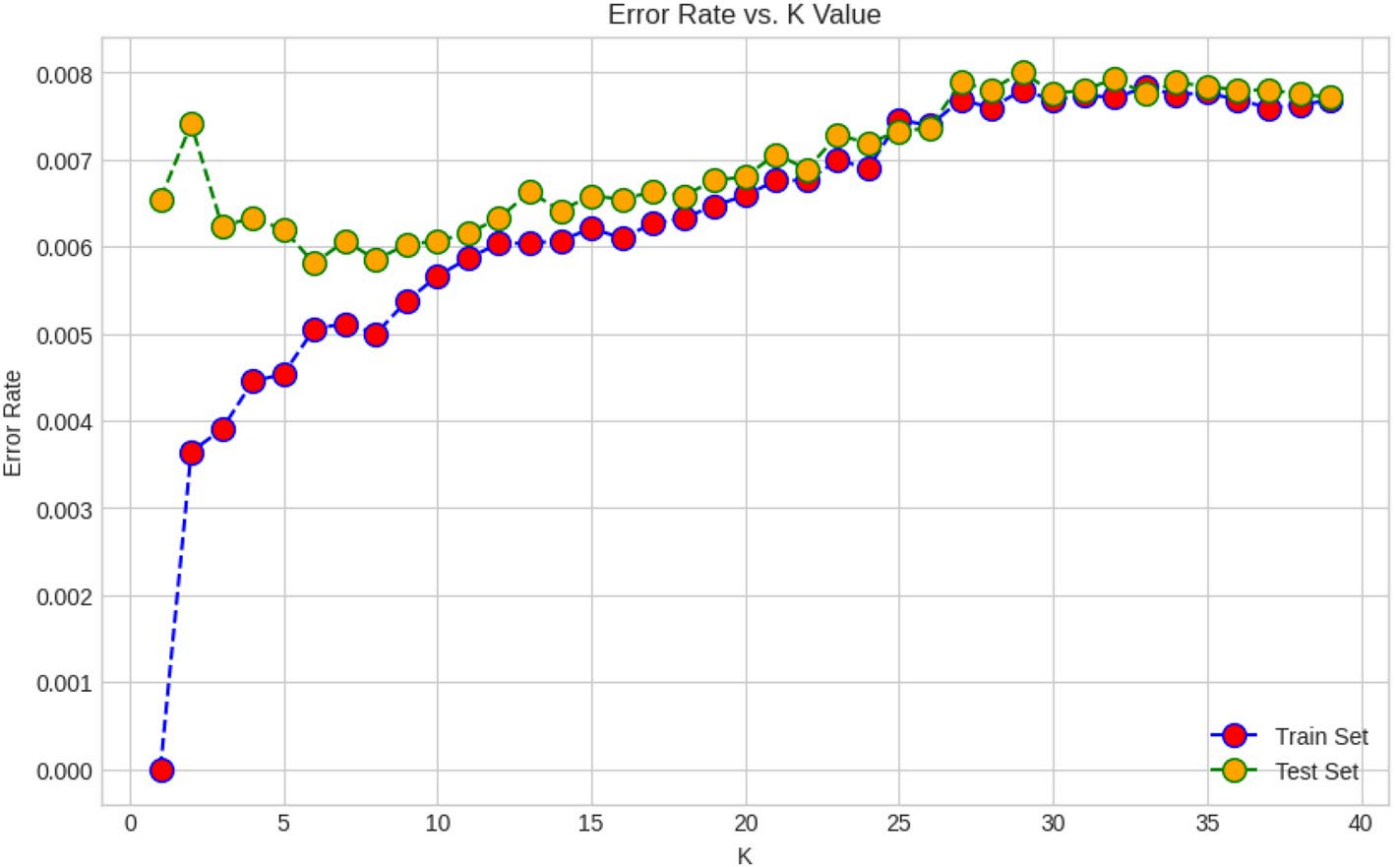
Error rate versus K plot for training and testing data.

In K-Nearest Neighbours (KNN) classification, it is very important to find the right amount of neighbours (K). To do this, we carefully plotted the error rate against K for both the training and testing datasets. Our goal was to find the best number that minimizes errors while reducing the risks of overfitting and underfitting. The error rate versus K plot (Fig. 12) showed that K = 10 was the best choice because it had the lowest error rate for both the training set and the test set.

The algorithms 3 and 4, respectively, represent the SVM and KNN classifiers. This is a multi-class classification issue, including three classes: class 1 for negative prostate test results, class 2 for suspicious results, and class 3 for non-suspicious anomalies. The confusion matrix between testing data and predictions was then examined. Finally, the classification report for the performance analysis of several proposed strategies is prepared.
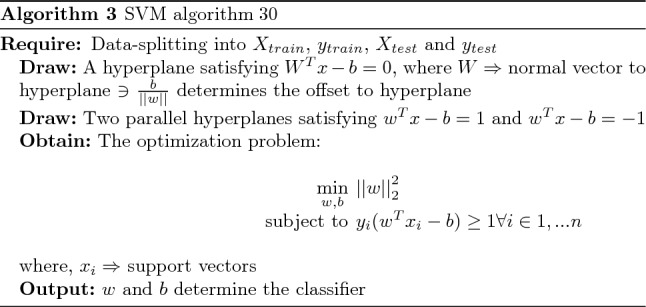

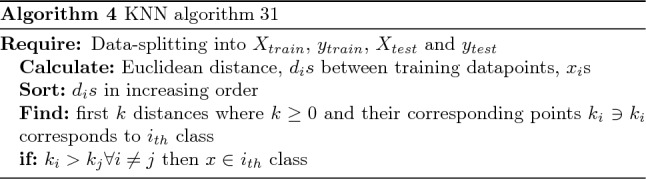


## Result and discussion

**Figure 13:**
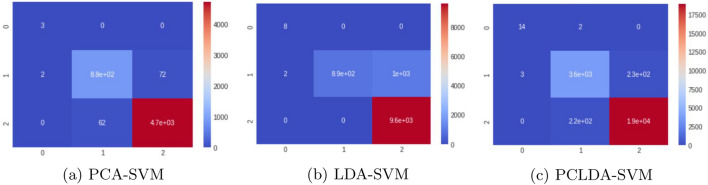
Heatmaps using SVM classifiers for the PLCO screening dataset.

**Figure 14:**
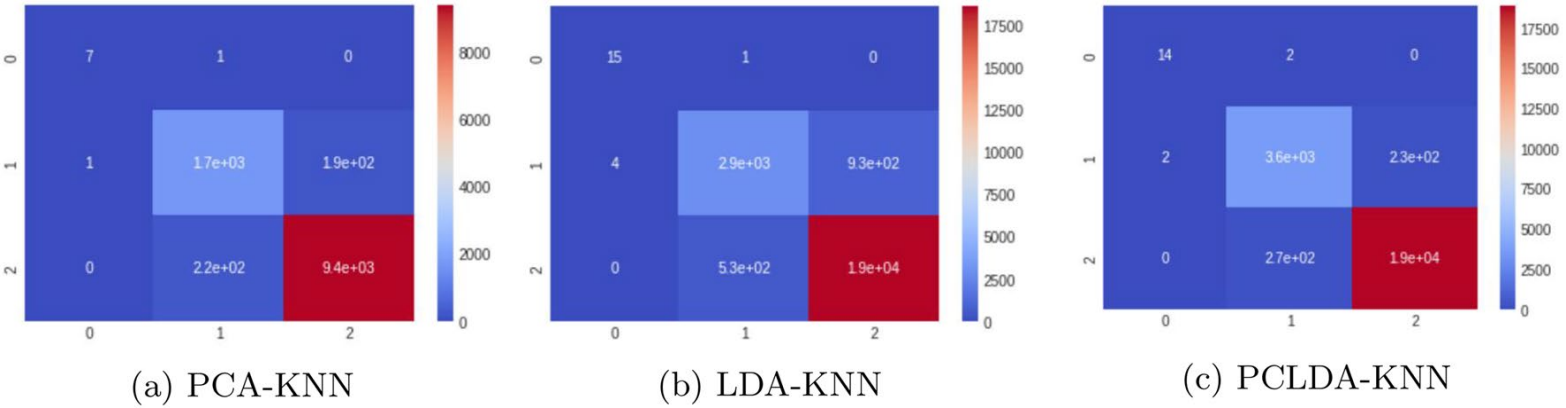
Heatmaps using KNN classifiers for the PLCO screening dataset.

We use 1,77,314 samples from the PLCO screening dataset for testing, and after data preprocessing, the sample size is decreased to 57,698. There are 46,158 samples for the training phase and 11,540 for the testing phase. Our model is evaluated using a dimension reduction method and classifiers. Figures 13 and 14 shows the heatmaps using different classifier models (Table 2).

**Figure 15:**
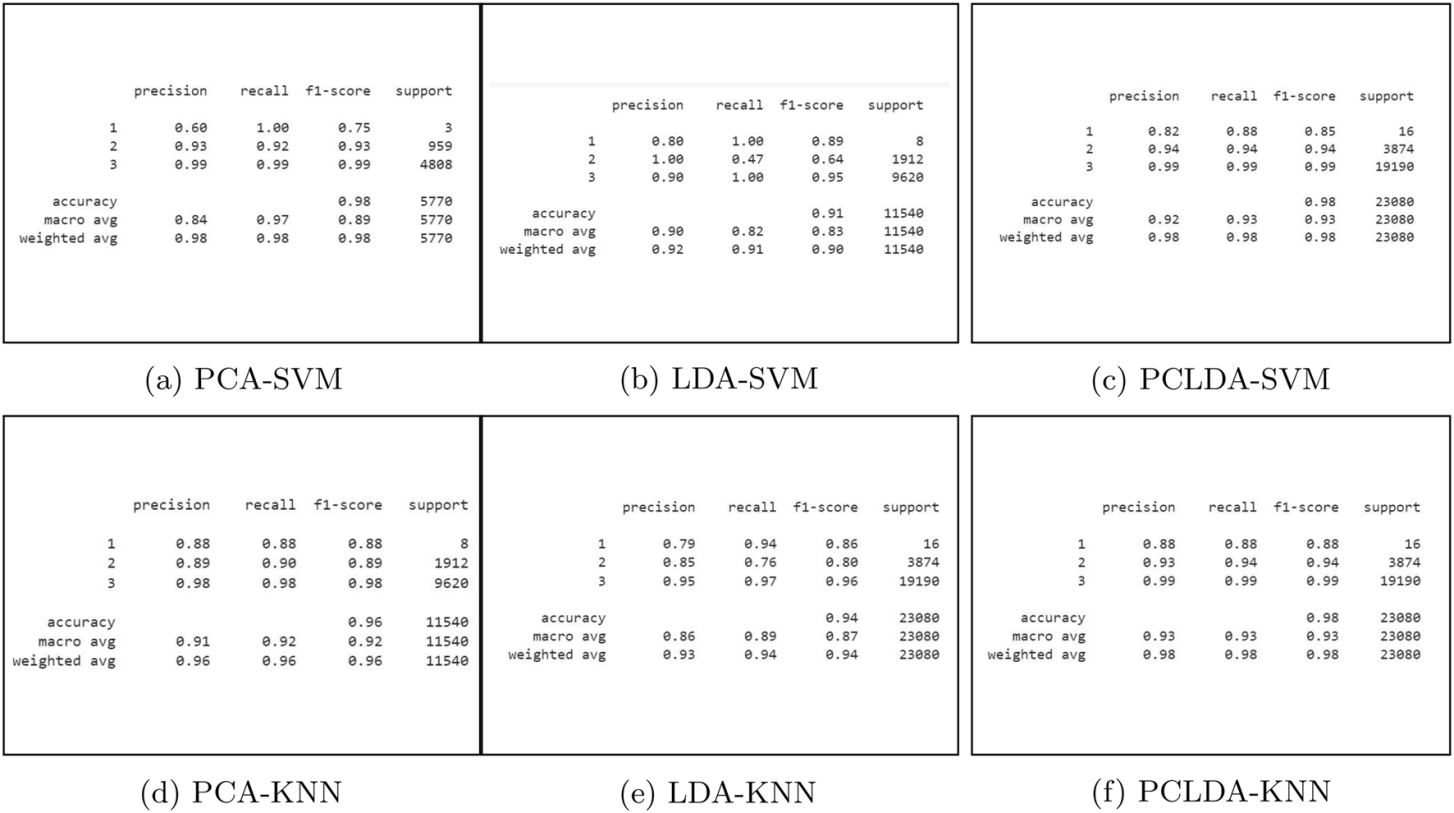
Classification report using SVM and KNN classifiers for the PLCO screening dataset.

After SVM and KNN classifiers were used, the confusion matrices for the training values and the predicted values were evaluated. Figure 15a–f illustrate the classification reports used to assess the models. From the Confusion Matrix of testing samples, accuracy, precision, sensitivity, and specificity are computed. The diagonal members of the Confusion matrix indicate correct predictions by the classifier when assessing performance measures. These components are further subdivided into accurately labelled True Positive (TP) and True Negative (TN) categories. False Negative (FN) and False Positive (FP) are non-diagonal elements for classes that have been erroneously labelled. Each classification model’s accuracy, precision, and sensitivity have been determined and summarized in Table 3. The following are definitions for Accuracy (ACCY), Sensitivity (SENS), Specificity (SPECY), F1 Score (FSC), and Precision (PRES):

8 $${} ACCY=\frac{TP+TN}{TP+TN+FP+FN}$$

9 $${} SENS=\frac{TP}{TP+FN}$$

10 $${} SPECY=\frac{TN}{TN+FP}$$

11 $${} PRES=\frac{TP}{TP+FP}$$

12 $${} FSC=\frac{2TP}{2TP+FP+FN}$$

In addition to these classification metrics, some additional classification coefficients such as Matthews Correlation Coefficient (MCC), kappa coefficients are error rates are evaluated for PCLDA-SVM and PCLDA-KNN models. Kappa coefficient is a statistical measure of inter-rater agreement between two annotators or classifiers. It considers observed agreement (Po) and expected agreement by chance (Pe). Error rate is a simple measure of classification error, representing the proportion of misclassified instances in a dataset. These coefficients are defined as:

13 $${} MCC = \frac{{TP \times TN - FP \times FN}}{{\sqrt{{(TP + FP) \times (TP + FN) \times (TN + FP) \times (TN + FN)}}}}$$

14 $${} Kappa = \frac{{Po - Pe}}{{1 - Pe}}$$

15 $${} Error Rate = \frac{{FP + FN}}{{TP + TN + FP + FN}}$$

**Figure 16:**
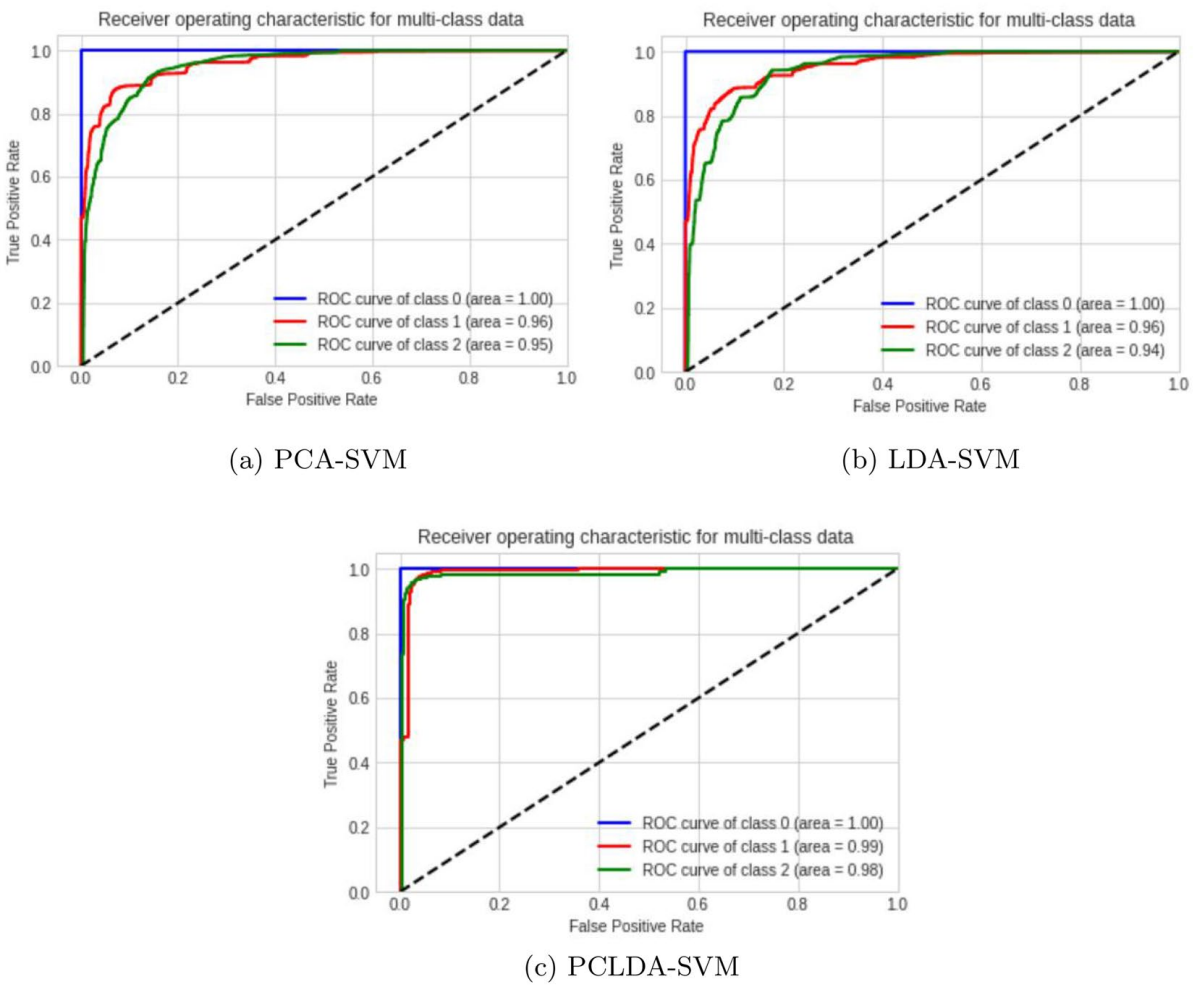
ROC plot for SVM classifier using PCA, LDA and PCLDA dimension reduction techniques.

Accuracy of Receiver Operating Characteristic (ROC Curve (ROC-AUC) is utilized as a basic graph for assessing various diagnostic tests in biomedical research for analyzing the performance in classification issues and different prediction models. Consequently, the ROC-AUC plot contains True positive rate (TPR) and False positive rate (FPR) parameters, which are measures of performance in the positive and negative portions of the sample, respectively. ROC curves for each classifier model for multiple classifications are shown in Fig. 16.

**Table 2:** Performance analysis using different classification models.

Classification model	Accuracy (%)	Precision	Sensitivity (%)	Specificity (%)	F1 score
PCA-SVM	97.64	0.84	96.7	97.6	0.89
LDA-SVM	91.17	0.90	82.34	90.71	0.83
PCLDA-SVM	97.99	0.92	92.83	97.65	0.93
PCA-KNN	96.44	0.91	91.93	95.58	0.92
LDA-KNN	93.64	0.86	88.93	93.7	0.87
PCLDA-KNN	97.83	0.93	93.39	97.86	0.92

The PCLDA-SVM model achieves an accuracy rate of 97.99%, which is the highest among all the classification models evaluated in the study. This indicates that the model correctly classifies 97.99% of the samples in the dataset. Comparing the other models, the PCA-SVM model achieves an accuracy rate of 97.64%, the LDA-SVM model achieves an accuracy rate of 91.17%, the PCA-KNN model achieves an accuracy rate of 96.44%, the LDA-KNN model achieves an accuracy rate of 93.64%, and the PCLDA-KNN model achieves an accuracy rate of 97.83%.

The precision of the PCLDA-SVM model is 0.92, indicating that it correctly identifies 92% of the true positive cases out of all the predicted positive cases. The sensitivity (also known as recall or true positive rate) of the PCLDA-SVM model is 92.83%, indicating that it correctly identifies 92.83% of the actual positive cases. The specificity (also known as true negative rate) of the PCLDA-SVM model is 97.65%, indicating that it correctly identifies 97.65% of the actual negative cases. The F1 score of the PCLDA-SVM model is 0.93, which is the harmonic mean of precision and sensitivity. It represents the balance between precision and recall and provides an overall measure of the model’s performance.

The MCC value for the PCA-LDA-SVM classification model is 0.9462, indicating a strong correlation between the predicted and actual labels. The Kappa coefficient, which measures the agreement between predicted and actual labels while taking chance into consideration, is 0.9461, indicating a high level of agreement beyond random chance. The classification model’s error rate is 0.0163, indicating a low rate of misclassification.

Based on these evaluation metrics, the PCLDA-SVM model demonstrates superior performance compared to other models in terms of accuracy, precision, sensitivity, specificity, and F1 score. The high accuracy rate of 97.99% indicates that the model is effective in distinguishing between cancerous and non-cancerous cases, and the high precision, sensitivity, specificity, and F1 score further validate its robust performance. Therefore, quantitatively, the PCLDA-SVM model stands out as the most accurate and reliable model for prostate cancer screening in this study.

**Table 3:** Classification coefficients for PCLDA-SVM and KNN models.

Classification model	MCC	Kappa coeff	Error rate
PCLDA-SVM	0.946	0.946	0.016
PCLDA-KNN	0.98	0.98	0.006

## Conclusion

A malignant condition, like prostate cancer, may be detected and treated with the use of screening findings. The National Cancer Institute (NCI)-approved PLCO dataset from the CDAS study is used. 80 columns or features and 177,314 occurrences were present. We only selected 13 essential characteristics, which were further narrowed down using the PCLDA approach. The collected dataset was then separated into three classes-Class 1 for negative results, Class 2 for abnormal-suspicious events, and Class 3 for abnormal non-suspicious-and utilised for classification. The recommended therapy may be determined using the expected values. In this multi-class classification, the SVM and KNN classifiers were used, and the PCLDA-SVM classification model demonstrated the greatest performance with an accuracy of 98%.

The study’s findings have significant implications for improving diagnostic accuracy and patient outcomes in prostate cancer screening. The PCLDA-SVM model’s higher performance shows that it may be a useful tool for medical practitioners in precisely identifying cases of prostate cancer. The methodology can result in prompt interventions, proper treatment plans, and improved patient outcomes by decreasing the rate of misdiagnosis and offering results that are more reliable. The paper also emphasizes the potential of integrating potent classification algorithms like SVM and KNN with dimensionality reduction approaches like PCA and LDA. The combination of these methods enables a thorough investigation of the data, allowing for the discovery of pertinent features and improving the categorization procedure. This combination of methods can improve the models’ performance and accuracy, leading to more successful prostate cancer screening.

In conclusion, the study’s findings emphasize the effectiveness of the PCLDA-SVM model for prostate cancer screening. By achieving high accuracy, precision, sensitivity, specificity, and F1 score, the model has the potential to significantly improve diagnostic accuracy, leading to better patient outcomes and a more efficient healthcare system. The integration of dimensionality reduction techniques with advanced classification models opens up new avenues for enhancing the accuracy and reliability of diagnostic models in various medical domains.

Future research in prostate cancer screening should consider exploring alternative dimensionality reduction techniques, such as t-SNE or autoencoders, to enhance the current study’s findings. Additionally, investigating the proposed approach in larger and more diverse datasets can provide a better understanding of its generalizability and performance across different populations. Integrating additional features, such as imaging data or biomarkers, could improve the accuracy and predictive power of the models. Prospective studies in a clinical setting are needed to evaluate the feasibility and impact of the proposed approach on patient outcomes. It is important to address limitations, such as including more patient characteristics and conducting cross-validation or external validation, to enhance the reliability and applicability of the findings. These avenues of research have the potential to advance prostate cancer screening and improve diagnostic accuracy in clinical practice.

## Data Availability

The Prostate, Lung, Colorectal, and Ovarian (PLCO) Cancer Screening Trial data were made available to the study’s author by the National Cancer Institute. The CDAS (Cancer Data Access System) specific project number is PLCO-934. A data transfer contract was also made between the study’s author and the NCI, reiterating their dedication to using ethical data handling procedures. The Agreement is accessible for review upon request. The Cancer Data Access System at the following website: https://cdas.cancer.gov/approved-projects/3475/ contains the datasets used in the current investigation.

## References

[1] Phan T, Crook SM, Bryce AH, Maley CC, Kostelich EJ, Kuang Y (2020). Mathematical modeling of prostate cancer and clinical application. Appl. Sci..

[2] Soronen V, Talala K, Raitanen J, Taari K, Tammela T, Auvinen A (2021). Digital rectal examination in prostate cancer screening at PSA level 3.0–3.9 ng/ml: Long-term results from a randomized trial. Scand. J. Urol..

[3] Koshkin VS, Patel VG, Ali A, Bilen MA, Ravindranathan D, Park JJ, Kellezi O, Cieslik M, Shaya J, Cabal A (2021). Promise: A real-world clinical-genomic database to address knowledge gaps in prostate cancer. Prostate Cancer Prostatic Dis..

[4] Dubey P, Kumar S (2022). Higher-order sliding mode control for androgen deprivation therapy. J. Electr. Eng. Technol..

[5] Society, A.C. Cancer Facts Statistics. https://www.cancer.org/research/cancer-facts-statistics/all-cancer-facts-figures/cancer-facts-figures-2022.html (2022)

[6] Teoh JY, Hirai HW, Ho JM, Chan FC, Tsoi KK, Ng CF (2019). Global incidence of prostate cancer in developing and developed countries with changing age structures. PLoS ONE.

[7] Robin TP, Geiger CL, Callihan EB, Kessler ER (2021). Prostate cancer in older adults: Risk of clinically meaningful disease, the role of screening and special considerations. Curr. Oncol. Rep..

[8] Hulsen T (2019). An overview of publicly available patient-centered prostate cancer datasets. Transl. Androl. Urol..

[9] Gelfond JA, Hernandez B, Goros M, Ibrahim JG, Chen M-H, Sun W, Leach RJ, Kattan MW, Thompson IM, Ankerst DP (2022). Prediction of future risk of any and higher-grade prostate cancer based on the PLCO and SELECT trials. BMC Urol..

[10] Bibault J-E, Hancock S, Buyyounouski MK, Bagshaw H, Leppert JT, Liao JC, Xing L (2021). Development and validation of an interpretable artificial intelligence model to predict 10-year prostate cancer mortality. Cancers.

[11] Bilal A, Zhu L, Deng A, Lu H, Wu N (2022). Ai-based automatic detection and classification of diabetic retinopathy using u-net and deep learning. Symmetry.

[12] Bilal A, Sun G, Mazhar S, Imran A, Latif J (2022). A transfer learning and u-net-based automatic detection of diabetic retinopathy from fundus images. Comput. Methods Biomech. Biomed. Eng. Imaging Vis..

[13] Bilal, A., Sun, G., Mazhar, S., & Imran, A. Improved grey wolf optimization-based feature selection and classification using CNN for diabetic retinopathy detection. In: Evolutionary Computing and Mobile Sustainable Networks: Proceedings of ICECMSN 2021, 1–14. Springer (2022)

[14] Bilal A, Sun G, Li Y, Mazhar S, Khan AQ (2021). Diabetic retinopathy detection and classification using mixed models for a disease grading database. IEEE Access.

[15] Bilal A, Sun G, Li Y, Mazhar S, Latif J (2022). Lung nodules detection using grey wolf optimization by weighted filters and classification using CNN. J. Chin. Inst. Eng..

[16] Bilal A, Sun G, Mazhar S, Junjie Z (2021). Neuro-optimized numerical treatment of HIV infection model. Int. J. Biomath..

[17] Bilal A, Shafiq M, Fang F, Waqar M, Ullah I, Ghadi YY, Long H, Zeng R (2022). IGWO-IVNet3: DL-based automatic diagnosis of lung nodules using an improved gray wolf optimization and inceptionnet-V3. Sensors.

[18] Bilal A, Sun G, Mazhar S (2021). Finger-vein recognition using a novel enhancement method with convolutional neural network. J. Chin. Inst. Eng..

[19] Preetha R, Jinny SV (2021). Early diagnose breast cancer with PCA-LDA based FER and neuro-fuzzy classification system. J. Ambient Intell. Humaniz. Comput..

[20] Alshareef AM, Alsini R, Alsieni M, Alrowais F, Marzouk R, Abunadi I, Nemri N (2022). Optimal deep learning enabled prostate cancer detection using microarray gene expression. J. Healthc. Eng..

[21] Akinnuwesi, B.A., Olayanju, K.A., Aribisala, B.S., Fashoto, S.G., Mbunge, E., Okpeku, M. & Owate, P. Application of support vector machine algorithm for early differential diagnosis of prostate cancer. Data Sci. Manag. (2022)

[22] Adiwijaya WU, Lisnawati E, Aditsania A, Kusumo DS (2018). Dimensionality reduction using principal component analysis for cancer detection based on microarray data classification. J. Comput. Sci..

[23] Hun, C.C., Yazid, H., Safar, M.J.A., & Ab Rahman, K.S. Comparison between k-nearest neighbor (KNN) and decision tree (DT) classifier for glandular components. In: Proceedings of the 11th International Conference on Robotics, Vision, Signal Processing and Power Applications, 292–297 (2022). Springer.

[24] System, C.D.A. Application and Analysis of Mathematical Tools in Biological Model. National Cancer Institute (2022)

[25] Zhao, N., Mio, W., & Liu, X. A hybrid PCA-LDA model for dimension reduction. In: The 2011 International Joint Conference on Neural Networks, 2184–2190 (2011). 10.1109/IJCNN.2011.6033499

[26] Yang J, Yang J-Y (2003). Why can LDA be performed in PCA transformed space?. Pattern Recognit..

[27] Hasan BMS, Abdulazeez AM (2021). A review of principal component analysis algorithm for dimensionality reduction. J. Soft Comput. Data Min..

[28] Perera M, Mirchandani R, Papa N, Breemer G, Effeindzourou A, Smith L, Swindle P, Smith E (2021). PSA-based machine learning model improves prostate cancer risk stratification in a screening population. World J. Urol..

[29] Rustam Z, Angie N (2021). Prostate cancer classification using random forest and support vector machines. J. Phys. Conf. Ser..

[30] Liu X, He W (2022). Adaptive kernel scaling support vector machine with application to a prostate cancer image study. J. Appl. Stat..

[31] Rani, S. *et al.* Comparative analysis of breast and prostate cancer prediction using machine learning techniques. In *International Conference on Innovative Computing and Communications, Proceedings of ICICC 2022* Vol. 1, 643–650 (Springer, Singapore, 2023).

